# Operations on the Heart

**Published:** 1918-10

**Authors:** 


***Immediate Operations for Wounds of the Heart from Projectiles of War.*** H. CONTANTINE and M. VIGOT. *Revue de Chirurgie,* September-October, 1917.

Emergency operations on the heart shortly after injury are very uncommon. Every case of injury to the chest should be examined by X-ray for immobility of the cardiac shadow which is characteristic of penetrating wounds of the heart. This report contains four operated cases with two deaths, operations being within 24 hours.

*Case I.* Suture of wound of posterior aspect of left ventricle after luxating heart. Organ steadied with left hand and three sutures passed. Ether, anesthesia, cure.

*Case II.* Suture of penetrating wound of left auricle. Cure.

*Case III.* Combined abdominal cardiac injury. Wretched condition prevented operation. Death. Autopsy showed a small projectile in apex of heart.

*Case IV.* Complicating injuries of arm and of thigh, hemothorax, with symptomless injury to heart. Shell fragment traversed left ventricle wall apex and lodged in wall of right ventricle. Death.

The author recommends making a trap-door incision of chest wall with hinge to the left, including three costal cartilages and sternum, so as to get a satisfactory exposure.

